# Supplementary material for: TurboID-Based IRE1 Interactome Reveals Participants of the Endoplasmic Reticulum-Associated Protein Degradation Machinery in the Human Mast Cell Leukemia Cell Line HMC-1.2
Source: Cells. 2024 Apr 25;13(9):747. doi: 10.3390/cells13090747 (PMC11082977; doi:10.3390/cells13090747)
Supplement: Supplementary file 1 [file cells-13-00747-s001.zip › Supplementary Table S1 and Figures.pdf]

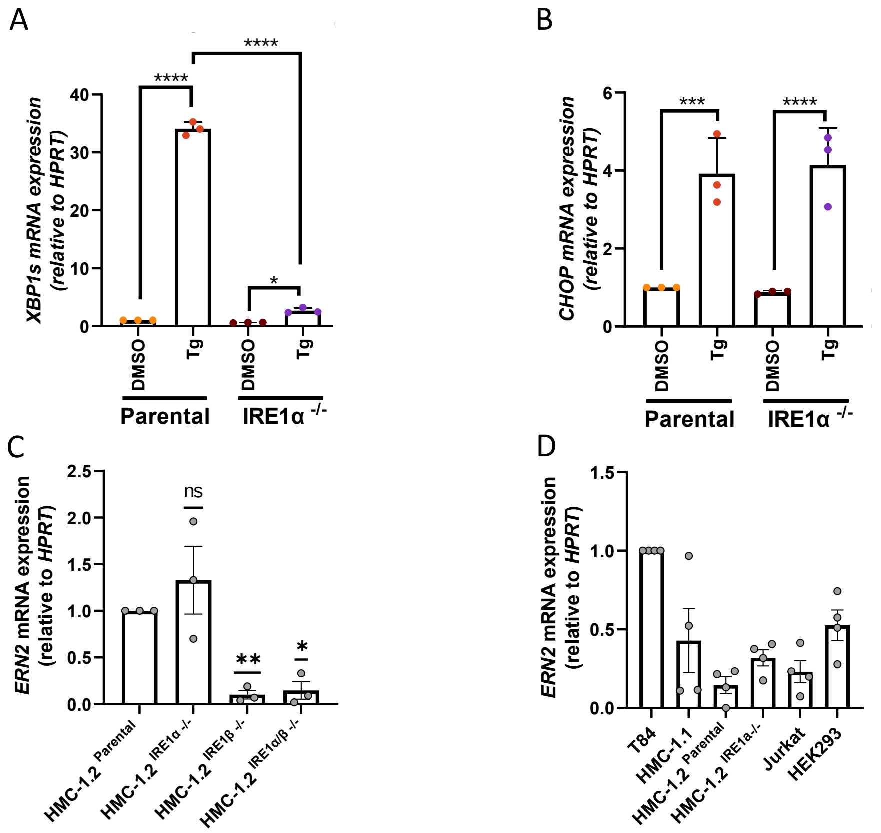

Figure S1: UPR activation in parental HMC-1.2 and IRE1α or IRE1β deficient cell lines.
(A-B) Parental and IRE1 KOs HMC-1. 2 cells were treated with vehicle (DMSO), 100 nM or thapsigargin (Tg) for 3 hr. *XBP1s* (**A**), *CHOP* (**B**) mRNA expression was evaluated by RT-qPCR and normalized to *HPRT* (n=3). (**C**) Expression levels of *ERN2* (IRE1β) in different human mast cell and the cancer cell lines HEK293 and Jurkat. Comparison between expression levels of *ERN2* in human mast cell_and non-mast cell lines. *HPRT* was used as a housekeeper. The expression values represent the mean of biological replicates (n=4). Data shown are mean ± SD. (**D**) RT-qPCR analysis of *ERN2* transcript expression in HMC-1.2^{parental} and IRE1 KOs HMC-1.2. The mRNA expression was evaluated by real-time quantitative PCR, normalized to *HPRT* and presented as mean +SD, n= 3. Data shown are mean ± SD. Significance levels are based on the One-Way ANOVA test. *$p < 0.05$, **$p < 0.01$, ***$p < 0.001$, ****$p < 0.0001$.

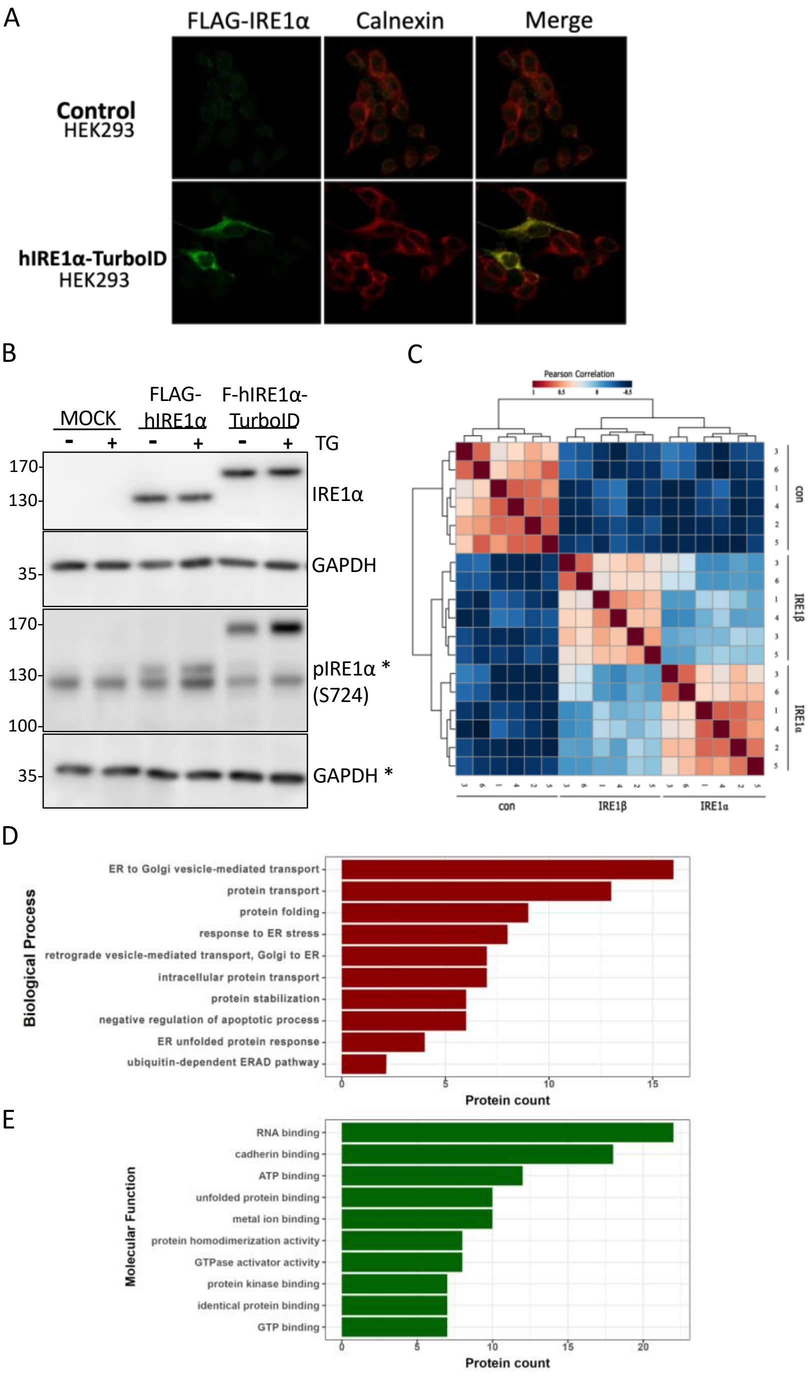

Figure S2: Characterization of the IRE1α-TurboID fusion protein
(**A**) ER localization of IRE1α-TurboID was validated by confocal microscopy using HEK293 cells expressing IRE1α-TurboID. Cells were stained with anti-Calnexin (red) and anti-FLAG-tag (green), and images were taken at 488 nm and 647 nm. Scale bar: 20 µm.
(**B**) Immunoblot of stably expressed MOCK-EV, FLAG-IRE1α-WT, or FLAG-IRE1α-TurboID (F-hIRE1α-TurboID). Cells were treated with 100 nM Tg or the solvent DMSO for 4 h, subsequently lysed and cell lysates were subjected to SDS-PAGE and Western blotting. IRE1α and phosphorylation were detected by anti-IRE1α and anti-pIRE1α (S724) antibodies. GAPDH served as a loading control. (**C**) Pairwise comparison correlation matrix heatmap between LFQ intensities of interacting proteins from TurboID cell lines. Except for the control sample, there is a high correlation of around 0.5. (D-E) The GO enriched terms of top overrepresented proteins in biological processes (**D**) and molecular function (**E**).

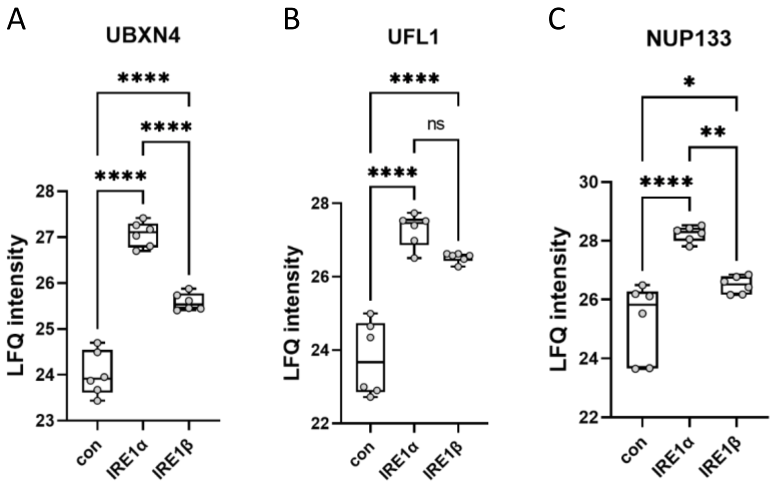

Figure S3: IRE1α interactions in HMC-1.2.
Boxplot depicts LFQ values of the LC/MS TurboID detected interactors UBXN4 (**A**), UFL1 (**B**) and NUP133 (**C**), respectively.

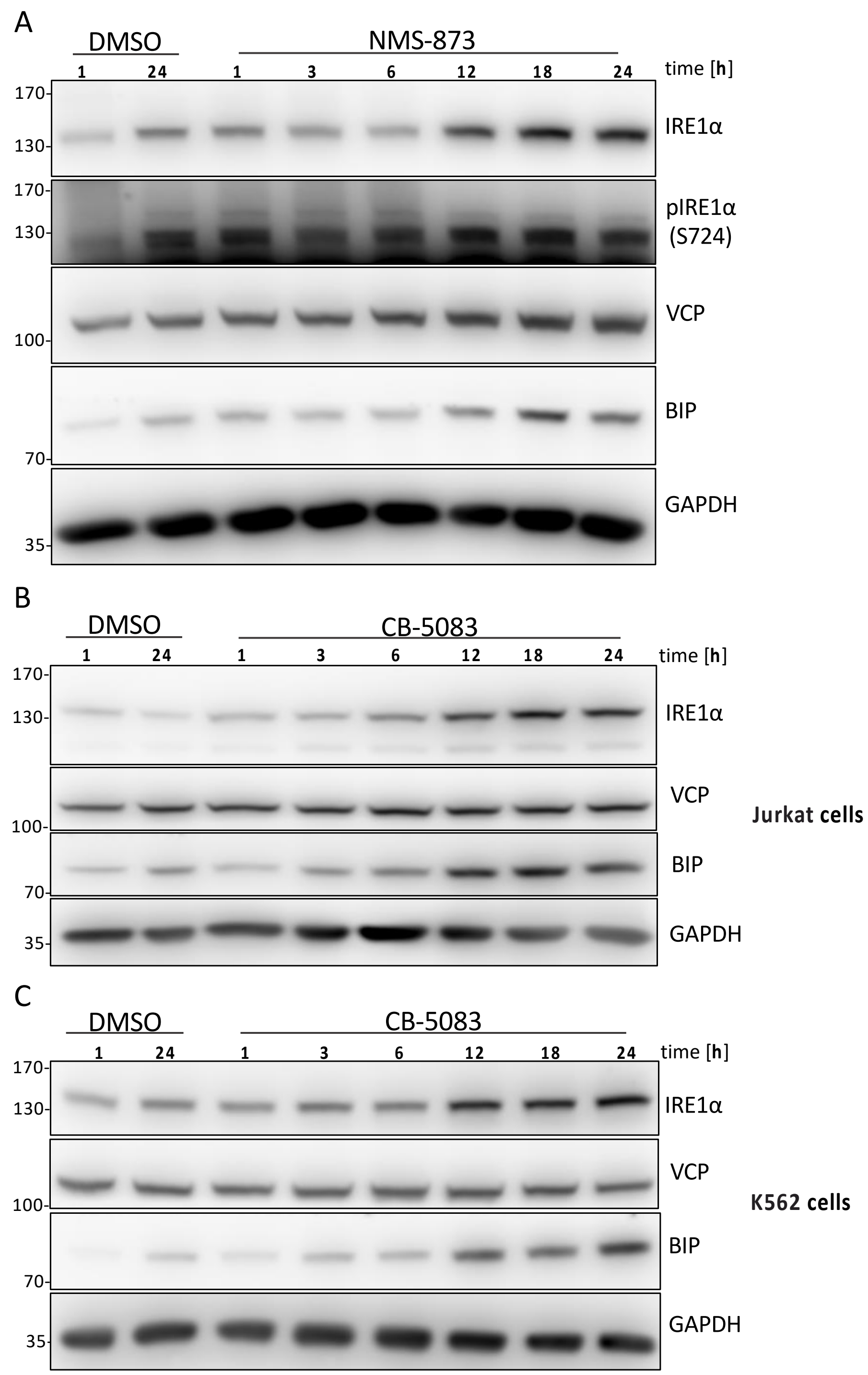

Figure S4: VCP inhibition stimulates UPR activation and IRE1α expression
(**A**) HMC-1.2 cells were treated with vehicle (DMSO) or 1 μM NMS-873 for the indicated time points. The phosphorylation of IRE1α (S724) and the expression of IRE1α, VCP and BIP were detected on Western blots. GAPDH served as loading control. (**B**) Jurkat and (**C**) K562 cells were treated with vehicle (DMSO) or 1 μM CB-5803 for the indicated time points. The expression of IRE1α, VCP and BIP were detected on Western blots. GAPDH served as loading control.

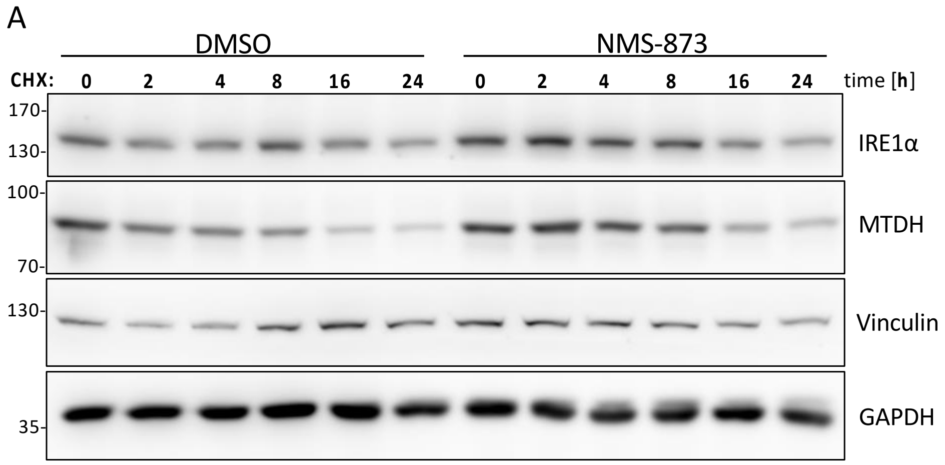

Figure S5: VCP inhibition by NMS-873 stabilizes IRE1*α* and MTDH
(**A**) Cycloheximide (CHX) chase assay in the presence of the VCP inhibitor NMS-873 (1μM) or the solvent DMSO. CHX treated HMC-1.2 cells were lysed at indicated time points and IRE1α and MTDH were detected on Western blots. Vinculin served as loading control.

Suppl. Table S1: Relevant GO terms with respect to biological processes and molecular functions including proteins detected and analyzed in this study

**Biological process**	**Symbol**
ER to Golgi vesicle-mediated transport	ARFGAP3, BCAP31, COPA, COPB1, COPB2, COPE, MIA2, MIA3, PREB, SCFD1, SEC16A, SEC22B, STX5, VAPA, VAPB, VCP, YKT6
Protein transport	ARFGAP3, BCAP31, COPA, COPB1, COPB2, KTN1, MIA3, NDC1, PTPN1, RAB3GAP2, SEC22B, STX5, USE1, VCP, YKT6
Protein folding	AIP, CDC37, DNAJC1, FKBP8, HSP90AB1, NUDC, NUDCD2, PTGES3, ST13, TTC1, UGGT1, UNC45A
Response to ER stress	ATP2A2, BCAP31, CDK5RAP3, DDRGK1, **ERN1**, SEC16A, TMX1, UFL1
Retrograde vesicle-mediated transport, Golgi to ER	AUP1, BCAP31, COPA, COPB2, COPE, NBAS, SCFD1, SEC22B, STX5, USE1, VCP, YKT6
Intracellular protein transport	ARFGAP3, COPA, COPB1, COPB2, PTPN1, RAB3GAP2, SCFD1, STX5
Protein Stabilization	CDC37, HSP90AB1, PIH1D1, PTGES3, RTN4, SEC16A
Negative regulation of apoptotic process	DDRGK1, FKBP8, HSP90AB1, MTDH, RPL10, UFL1
ER unfolded protein response	CDK5RAP3, **ERN1**, PTPN1, VAPB, VCP
Ubiquitin-dependent ERAD pathway	AUP1, UBXN4, VCP

**Molecular Function**	**Symbol**
RNA binding	ALDH18A1, ATP5F1A, CISD2, FBL, HSP90AB1, KTN1, LBR, LRRC59, MTDH ,PKM, PTPN1, RPL10, RPL9, RRBP1, RTN4, SEC63, SND1, SRP54, SRPRA, STIP1, SUGT1, VCP, ZC3HAV1
Cadherin binding	ARHGAP1, EMD (Emerin), HSP90AB1, KTN1, LRRC59, NUDC, PFKP, PKM, PTPN1, RAB11B, RTN4, SND1, STX5, UNC45A, VAPA, VAPB, YKT6, ZC3HAV1
ATP binding	ACSL3, ACSL5, ALDH18A1, ATP2A2, ATP5F1A, **ERN1**, HSP90AB1, KIT, PC, PFKP, PKM, VCP
Unfolded protein binding	AIP, CCDC115, CDC37, **ERN1**, HSP90AB1, NUDC, NUDCD2, PTGES3, TTC1, UGGT1
Protein homodimerization activity	CACYBP, CISD2, ERN1, HSP90AB1, KIT, PKM, RTN4, VAPB
Protein kinase binding	ACSL3, CDC37, CDK5RAP3, HSP90AB1, PIH1D1, PTPN1, UFL1
Identical protein binding	ALDH18A1, ERN1, FKBP8, HSP90AB1, PC, PFKP, VCP

The bait **ERN1** (IRE1α) is written in bold, detected and analyzed interaction partners are highlighted in yellow.
